# Organophosphorus Compounds and MAPK Signaling Pathways

**DOI:** 10.3390/ijms21124258

**Published:** 2020-06-15

**Authors:** Tahereh Farkhondeh, Omid Mehrpour, Constanze Buhrmann, Ali Mohammad Pourbagher-Shahri, Mehdi Shakibaei, Saeed Samarghandian

**Affiliations:** 1 Medical Toxicology and Drug Abuse Research Center (MTDRC), Birjand University of Medical Sciences (BUMS), Birjand 9717853577, Iran; farkhondeh2324@gmail.com (T.F.); omid.mehrpour@yahoo.com.au (O.M.); ali.pourbagher.shahri@gmail.com (A.M.P.-S.); 2 Rocky Mountain Poison and Drug Safety, Denver Health, Denver, CO 80204, USA; 3 Musculoskeletal Research Group and Tumour Biology, Chair of Vegetative Anatomy, Institute of Anatomy, Faculty of Medicine, Ludwig-Maximilian-University Munich, Pettenkoferstrasse 11, D-80336 Munich, Germany; constanze.buhrmann@med.uni-muenchen.de; 4 Healthy Ageing Research Center, Neyshabur University of Medical Sciences, Neyshabur 9318614139, Iran

**Keywords:** organophosphorus compounds (OPC), MAPK signaling pathway, oxidative stress, apoptosis, ERK, JNK, p38-MAPK

## Abstract

The molecular signaling pathways that lead to cell survival/death after exposure to organophosphate compounds (OPCs) are not yet fully understood. Mitogen-activated protein kinases (MAPKs) including the extracellular signal-regulated protein kinase (ERK), the c-Jun NH2-terminal kinase (JNK), and the p38-MAPK play the leading roles in the transmission of extracellular signals into the cell nucleus, leading to cell differentiation, cell growth, and apoptosis. Moreover, exposure to OPCs induces ERK, JNK, and p38-MAPK activation, which leads to oxidative stress and apoptosis in various tissues. However, the activation of MAPK signaling pathways may differ depending on the type of OPCs and the type of cell exposed. Finally, different cell responses can be induced by different types of MAPK signaling pathways after exposure to OPCs.

## 1. Introduction

There is evidence that organophosphate pesticides (OPs) contribute to the etiology of many diseases including neurodegenerative, cardiovascular, respiratory, gastrointestinal, diabetic, dyslipidemic, and kidney diseases [1]. In addition, oxidative stress usually acts as the main mechanism in the pathogenesis of various diseases. In particular, inflammatory stimuli can synergistically enhance the influence of oxidative stress in different tissues [2]. Several researchers have pointed out that there is a correlation between OPs and the activation of signaling pathways for oxidative stress through the production of reactive oxygen species (ROS) [3]. ROS leads to further tissue damage via induction of lipid peroxidation, protein oxidation, and cell apoptosis [4].

Oxidative stress-induced by OP exposure can stimulate the mitogen-activated protein kinase (MAPK) signaling pathway in the tissue [3]. MAPK modules are key players of intracellular signal transduction with major roles in several physiological processes [5]. There are three subfamilies of these serine/threonine kinases in mammals, including extracellular responsive kinases (ERKs), c-Jun N-terminal kinases (JNKs), and p38-MAPKs, which can be activated by various types of OPs [6]. Activation of ERK, JNK, and p38 kinase in response to oxidative stress via ROS production has both a survival-promoting and a proapoptotic effect [7].

The activation of phospholipase C, gamma (PLC-gamma), and the proto-oncogenic tyrosine-protein kinase c-Src (c-Src) phosphorylate Ras and Raf by ROS both directly and indirectly activates ERK signaling [8].

ERK, one of the main signaling cassettes of the MAPK signaling pathway, contributes to the modulation of meiosis and mitosis functions in differentiated cells. In mammals, ERK consists of ERK 1/2, ERK3/4, ERK5, ERK7, and ERK8 classes. The ERK family plays the main role in the regulation of cell growth cycles, survival, and differentiation under normal and pathological conditions [8]. Activated ERK has negative and positive influences on the regulation of ROS production indirectly through p22phox induction, which increases ROS production; Nrf2 activation subsequently increases the expression of anti-oxidant components [9]. In addition, ROS leads to the activation of the apoptosis-signal-regulating kinase 1 (ASK1) through the activation of thioredoxin (Trx) and protein phosphatase 2C cDNA (PP2C epsilon). They block the inhibition of ASK1 and induce PP2B activation that positively regulates ASK1. Finally, the activated ASK1 stimulates the JNK and p38-MAPK signaling pathways [10] and JNK signaling in response to oxidative stress promotes either survival or proapoptotic pathways in cells [10]. In addition, the JNK signaling pathway has been reported to induce cell survival through forkhead box (Fox) activation, an increase in antioxidant production, and SIRT1 activation leading to inhibition of the p53-dependent signaling pathway [10]. Controversially, the JNK signaling pathway induces apoptosis in the cytoplasm and nucleus by activation of proapoptotic molecules and by preventing the activation of antiapoptotic molecules in the cytoplasm [10]. Furthermore, the JNK signaling pathway in the cell nucleus induces the activation of several transcription factors, which leads to an increase in proapoptotic gene expression [10]. In addition, the stimulated p38-MAPK signaling pathway enters the cell nucleus and promotes phosphorylation of the transcription factors of the activating transcription factor 2 (ATF2) and the protein binding the cAMP response element (CREB) by activating mitogen- and stress-activated protein kinases 1 and 2 (MSK1/2) [11]. Subsequently, ATF2 and CREB induce the expression of several proapoptotic genes, which subsequently lead to apoptosis [11].

This review was intended to evaluate and show the influence of OPs on the activation of the MAPK signaling pathway by oxidative stress and the association of the activated pathway with various diseases.

## 2. General Characteristics of Organophosphorus Compounds

It has been shown that organophosphorus compounds (OPCs) are organic compounds consisting of a phosphoryl (P=O) bond or a thiophosphoryl (P=S) bond, and several OPCs with different chemical, physical, and biological properties are commercially used. Furthermore, most of them are dissolved in oils and only a little in water, and the classification of OPCs is quite complex. There is therefore no uniform classification system for them. Indeed, OPCs derived from phosphoric acid and phosphonic acid derivatives usually have anti-cholinesterase activity, whereas OPCs derived from phosphonic acid do not have this property [12]. Examples of OPCs are the following: 1. insecticides such as diazinon (DZN), malathion, parathion, chlorpyrifos (CPF), dichlorvos, fenthion, and ethion; 2. nerve gases such as sarin, soman, tabun, and VX; 3. ophthalmic agents such as isofluorophate and echothiophate; 4. anti-helminthic agents such as trichlorfon; and 5. herbicides such as tribufos (DEF) and merphos [13]. To qualify OPCs as an insecticide, it is necessary to be effective at low doses and OPCs should also have a low toxic effect on humans and animals. Most OPCs are in liquid form with different vapour pressures at 20 °C. They are used as liquid sprays or granules for agricultural purposes, and they are broken down by the hydrolysis process, which produces water-soluble products [14]. The toxicity of most OPCs is high, oral LD_50_ for humans is between 0.5 and 5 g/kg, and oral or respiratory exposure to 10 to 200 mg parathion is fatal for adults [12]. However, it has been suggested that water-soluble products are nontoxic at concentrations for practical applications.

The estimated lethal doses of DZN, malathion, and parathion in humans are 25 g, 60 g, and 10 to 200 mg respectively [15]. Interestingly, it has been reported that, in developing countries, poisoning by OPCs is one of the most common types of poisoning leading to hospitalization [16]. The traditional approach suggested that the clinical symptoms of acute OPC intoxication were related to their effects on muscarinic, nicotinic, and CNS receptors. The general exposure to OPCs was usually through the consumption of food residues or the working environment. Moreover, OPCs can enter the body through ingestion, inhalation, or the skin. The metabolism of OPCs is usually by oxidation, hydrolysis, demethylation, and/or glucuronidation, and the oxidation of OPCs can lead to the formation of products with lower or higher toxic activity. It is known that the majority of OPCs is rapidly degraded and that this compound is mainly excreted in urine and, to a lesser extent, in feces. Although OPCs are not stored in the body for long, some of them are very lipophilic and are deposited in the fatty tissue for several days. Moreover, the binding of OPCs to acetylcholinesterase (AChE) in plasma and red blood cells are the main mechanisms of acute and chronic toxicity in the nervous system, and the activity of cholinesterase in red blood cells indicates the severity of toxicity of OPCs [16].

Most symptoms of OP intoxication are caused by the stimulation of several muscarinic receptors, and tachycardia and hypertension are sometimes observed in acute intoxications associated with the cholinergic effects of OPs on the CNS, sympathetic ganglion synapses, or the adrenal medulla [17]. A few numbers of OPs lead to an “intermediate syndrome” and a delayed neuropathy without inhibitory effect on AChE activity. Importantly, the nervous, cardiovascular, and respiratory systems are the main target organs for OPCs and it has been found that OPs can also inhibit the activity of other esterases [18].

Many of the OPs have no tumorigenic effect in animals, but some of them could induce cancer in experimental models [19,20]. Teratogenic and mutagenic effects have been found for some OP compounds. Some drugs, including antihistamines, phenothiazines, CNS depressants, theophylline, barbiturates, aminoglycosides, and parasympathomimetic agents, may exacerbate the toxicity of OPCs [21]. Indeed, in most poisonings, the interaction between organophosphate enzymes and enzymes that are both stable and treated occurs over a long time [21]. The inhibited enzyme can reactivate spontaneously, its rates depending on the tissue and the chemical group bound to the enzyme [21]. Further, OPCs are used as chemical warfare agents, which have stronger toxicity than insecticides. However, the use of OPCs in high concentrations can cause a toxic effect similar to chemical warfare agents [22].

## 3. Oxidative Effect of Organophosphate Pesticides is Mediated by MAPK Signaling

Oxidative stress is caused by a disturbance of the balance between oxidative and antioxidative systems, which leads to an impairment of the antioxidative defense [23]. It has been pointed out that oxidative stress leads to oxidation of lipids, proteins, and DNA, which causes various pathological states [23,24,25,26,27,28]. OPs induce oxidative damage by attacking one of the three main MAPK subfamilies, including ERK, JNK, and p38-MAPK kinase. In this context, it has been shown that acute exposure of mouse diaphragm muscle cells to dichlorvos (DDVP) induces oxidative stress, as evidenced by a decrease in the activities of AchE, Quinone oxidoreductase-1 (NQO-1), heme oxygenase 1 (HO-1), paraoxonase1 (PON1), catalase (CAT), superoxide dismutase (SOD), and Nrf2 protein expression as well as an increase in malondialdehyde (MDA) levels in the cells [28]. Similarly, it was observed that subacute exposure to DDVP induced liver injury in mice by oxidative stress by downregulation of *PON1* and *Nrf2* gene expression. The study showed that DDVP increased MDA levels and decreased glutathione (GSH), SOD, and CAT activities in the liver [29]. Furthermore, Jahan and colleagues [30] pointed out the effect of monocrotophos (MCP) on MAPK signaling in mesenchymal stem cells from human umbilical cord blood, suggesting that MCP increased ROS production by activating the ERK/AP-1 signaling pathway. In this context, it was shown that tris(1,3-dichloro-2-propyl)phosphate (TDCPP) reduced Nrf2, SOD1, and SOD2 expression in a dose-dependent manner in human umbilical cord endothelial cells (HUVECs) [31]. Further, there is evidence that CPF is involved in the impairment of oxidative stress signaling pathways, including MAPK signaling. Indeed, in *Drospphila melanogaster*, CPF increased the phosphorylation of p38-MAPK and JNK [32]. Shou and colleagues [33] investigated the effect of CPF on oxidative damage in PC12 cells, suggesting that CPF induced oxidative and apoptotic damage by the interfering Nrf2 signaling pathway, as evidenced by an increase in ROS and MDA values and a decrease in SOD values. In addition, OPCs exerted an inflammatory effect in the different tissues by inducing inflammatory mediators such as tumor necrosis factor alpha (TNF-α), interleukin-6 (IL-6), and IL-1β. It was found that the inflammatory and oxidative effects of OPCs are due to activation of the MAPK signaling pathway [34]. In fact, oxidative stress can induce the expression of inflammatory master transcription factors such as the nuclear transcription factor kappa-B (NF-κB), which are the most important regulatory factors in the induction of pro-inflammatory gene products and inflammation. It has been suggested that CPF induces apoptosis by the initial induction of oxidative stress in SH-SY5Y cells and that CPF stimulated molecular pathways including p38-MAPK, JNK and ERK, NF-κB, and TNF-α. Moreover, it has been shown that CPF also induced the activation of apoptotic pathways by increasing NF-κB. Therefore, oxidative damage of OPs is mainly mediated by stimulation of the MAPK signaling pathway [34].

## 4. Apoptotic Effect of Organophosphorus Compounds Is Mediated by MAPK Signaling

Apoptosis or programmed cell death is regulated also by the MAPK signaling pathways under stress conditions. Three conventional MAPK signaling pathways (ERKs, JNKs, and p38-MAPKs) play an important role in modulating the apoptotic pathway. Indeed, it was shown that activated JNKs and p38-MAPKs stimulate apoptosis cascades, while ERK1/2 plays an important protective role against apoptosis [35]. It has been suggested that the balance between growth factor-activated survival strategies and stress-activated death strategies affect cell death or survival. Numerous studies have pointed to the antiapoptotic effect of activated ERK1/2 and the proapoptotic effect of retained activated p38-MAPK and JNK. In this context, it has been shown that the MAPK and PI3K/Akt signaling pathway was upregulated by phoxim, leading to apoptosis and inhibition of protein synthesis in silkworms [36]. Esquivel-Sentíes et al. [37] evaluated the effects of diethyl dithiophosphate (DEDTP) on human CD4+ T lymphocytes. Indeed, in vitro exposure to DEDTP reduced the expression of CD25 and the concentrations of IL-2, which caused an impairment of cell proliferation. DEDTP stimulated the activation of ERK, JNK, and p38 as well as the nuclear factor of activated T cells (NFAT), resulting in a decrease in cell proliferation. Thus, OPCs are involved in the apoptotic signaling pathway by induction of the MAPK signaling pathway. Table 1 indicates OPC-induced several diseases mediated by MAPK signaling.

## 5. Organophosphorus Compound-Induced Neurodegenerative Diseases are Mediated by MAPK Signaling

Several members of the MAPK family, such as JNK, p38-MAPK, and ERK, are implicated in many neurodegenerative processes. The effects of OPCs on the MAPK signaling pathway-mediated neurodegenerative diseases have been reported in several scientific works of literature. OPCs disturb the balance between JNK, p38-MAPK, and ERK inducing apoptotic, oxidative, and inflammatory damages in neurons. Because exposure to CPF is more common among people, the majority of studies focused on the CPF neurotoxicity to study the impact of insecticides in the MAPK signaling pathways. In addition, it has been reported that CPF induces neuronal damage by activating three main pathways of MAPK including p38-MAPK, JNK, and ERK signaling. The effects of CPF on the MAPK pathways indicated that CPF has a major role in the induction of apoptosis, inflammation, and oxidative stress. The investigation on neurotoxic effects of CPF in PC12 cells indicated that treatment with 0, 25, 50, 100, and 200 μM CPF induced dose-dependently apoptosis via activating the p38-MAPK, JNK, and ERK, suggesting that activated p38-MAPK, JNK, and ERK signaling pathways acted as death signals and also activated caspase-3 and poly(ADP-ribose) polymerase (PARP) cleavage [38]. Additionally, the induction of apoptosis by CPF in the dopaminergic neurons of PC12 cells through activation of the ERK 1/2, JNK, and p38-MAPK via ROS production indicated its potential to produce oxidative stress via mitochondrial damage in neurodegenerative disorders [38]. The association between CPF exposure and Parkinson’s disease mostly has been investigated using SH-SY5Y cells. Treatment with 0, 25, 50, 100, and 200 μM CPF induced activation of p38-MAPK, JNK, and ERK, leading to an elevation in the expression of inflammatory genes including COX-2 and TNF-α. Additionally, CPF stimulated the nuclear translocation of NF-κB. Moreover, Rosiglitazone (RGZ), a peroxisome proliferator-activated receptor-gamma (PPAR-γ) agonist, was also used to prevent CPF-induced neuronal cell death and it was found that RGZ inhibited NF-κB expression through blocking p38-MAPK and JNK signaling activation [34]. CPF also caused PD by inducing MAPK-mediated translocation of dynamin-related protein 1 (Drp1) to the mitochondria and initiated mitochondrial fission and induced mitochondrial impairment that eventually caused mitochondrial membrane potential dissipation [39]. An elevation of phospho-Drp1 expression was observed in SH-SY5Y cells exposed to CPF [39].

It is known that Drp1 is necessary for the translocation of p53 into the mitochondria after CPF-increased ROS production. Blocking the Drp1 function-inhibited CPF caused mitochondrial-dependent apoptosis by interaction with p53 [35]. Ki et al. showed JNK- and p38-mediated expression of COX-2 in SH-SY5Y cells that were exposed to CPF [5]. They found further that the inhibitors of JNK and p38-MAPK improved CPF-increased COX-2 expression but that the inhibitor of ERK1/2 signal transmission had no effect on COX-2. Moreover, CPF has been hypothesized to cause neuronal apoptosis by increasing oxidative stress and COX-2 expression mediated by JNK and p38-MAPK signaling pathways. CPF also induced COX-2-mediated cytotoxicity regardless of the activation of the ERK1/2 signaling pathway [5]. In addition, chronic exposure to OPCs caused delayed neuropsychiatric disorders, and a mechanism underlying the delayed neurotoxicity induced by CPF can be the inhibition of ERK1/2 signaling activation. In this context, it was shown that phosphorylation of ERK1/2 during a 96-h exposure to 10 µM CPF occurred but that withdrawal after 48 h of exposure to this agent caused ERK1/2 signal inhibition, which delayed toxicity of CPF in primary rats hippocampal neurons [40].

Treatment with two activators of ERK1/2, such as nerve growth factor (NGF) and carbachol, inhibited the delayed neurotoxicity induced by CPF, which shows the influence of the inhibition of ERK1/2 phosphorylation on the delayed neurotoxicity caused by CPF [40]. Moreover, Caughlan et al. [41] have shown that CPF causes mitochondrial dysfunction and apoptosis in primary cortical neurons that have been cultured since the 17th day of embryonic life or in newborn rats. In this context, CPF increased the phosphorylation of ERK1/2 and of p38-MAPKs and CPF was unable to induce total JNK but activated a partial pool of JNK in the nucleus and induced c-Jun phosphorylation. The authors also suggested that the ERK1/2 and JNK signal activation induced apoptosis but that the activation of p38-MAPKs prevented apoptosis in cortical neurons exposed to CPF. Another CPF-induced developmental neurotoxicity is the disruption of cytoskeletal proteins, and neurite retraction in mouse N2a neuroblastoma cells is associated with a transient increase in NFH phosphorylation and ERK1/2 pathway activation [42]. It was reported that CPF exposure (5 mg/kg, daily) in substantia nigra (SN) in young adults with PND induced 11–14 dopaminergic neuronal damage in SN after activation of the inflammatory response by p65-NF-κB and p38-MAPK signaling pathways in the nigrostriatal system [43].

A study by Lazar et al. [44] showed a temporary increase in ERK1/2 activity and no modification to JNK in the hippocampus and cortex of male rats that were exposed to 80 μg/kg sarin after 6 h. After 24 h, an increase in the expression of Bax, a decrease in the ERKs, and an increase in the activity of JNK in the frontal cortex were observed. They suggested that the rapid increase in the activity of ERK1/2 without changing the JNK can show a mechanism of “first reaction” that temporarily inhibits apoptosis. Administration of sarin (0.4 mg/kg) or soman (4.0 mg/kg) to young rats increased the levels of tyrosine-phosphorylated proteins in the cytosol fraction of the brain, and JNK activation was found in the cytosol. In addition, JNK activation occurred after tyrosine kinase phosphorylation, which indicates that the activation of tyrosine kinase plays a major role in the neurotoxicity of Sarin and soman [45]. Pejchal and coworkers [46] have shown the expression of activated p38-MAPK signaling and activated MAPK transcription factors c-jun, c-myc, and elk-1 in cerebellar granula cells of rats after soman exposure, suggesting that, 14 days after soman poisoning, expression of p38-MAPK and c-myc was increased while c-jun and elk-1 expression did not change. Indeed, it has been suggested that delayed activation of p38-MAPK may be responsible for soman’s neurotoxic effects [46]. They also examined the expression of phosphorylated p38-MAPK and Elk-1, c-jun, and c-myc in the cerebellar Purkinje cells of rats after soman exposure [46]. Interestingly, an increase in activated p38-MAPK and c-myc expression was observed 2 weeks after exposure to soman while the expression of activated elk-1 and c-jun was not changed 14 days after poisoning. Chang [47] reported that bilateral injection of 10 nmol Mevinphos (Mev) into the rostral ventrolateral medulla (RVLM) of rats during brain stem death did not affect overall ERK1/2 but that phosphorylation of ERK1/2 in Thr202 and Tyr204 was stimulated. They further demonstrated the effect of Mevinphos (Mev, 10 nmol) on JNK and p38-MAPK in the rostral ventrolateral medulla (RVLM) of rats during experimental brainstem death, suggesting that Mev had no effect on the total amount of JNK, p38-MAPK, MAP2K4, and MAP2K6. However, Mev increased the phosphorylation of JNK in Thr183 and Tyr185, of p38-MAPK in Thr180 and Tyr182, of MAP2K4 in Ser257 and Thr261, and of MAP2K6 in Ser207 and Thr211 in RVLM during the pro-life phase of brain stem death. In addition, the phosphorylation of ATF-2 in Thr71 and of c-Jun in Ser73 in RVLM was also increased during this phase Chan et al. [48]. Furthermore, Chan et al. [49] have shown that Mev induced pro-life brainstem death via NO produced by NOS I in the Rostral Ventrolateral Medulla (RVLM), followed by stimulation of the soluble guanylyl cyclase/cGMP/PKG cascade. Further, it was shown that Bis (pinacolylmethyl) phosphonate (BPMP) induced the ERK signaling pathway to cause vacuolation in mitochondria in cultured rat astrocytes, and the authors suspected that the activated Ras/Raf-1/ERK signaling pathway was involved in modulating central cardiovascular function through NOS I/PKG upregulation [50].

## 6. Organophosphorus Compound-Induced Cardiovascular Diseases are Mediated by MAPK Signaling

It has been reported that cardiomyocyte survival and death are regulated by extracellular ligands, cytokines, and growth factors that bind to cell surface receptors and induce intracellular specific signal transduction pathways [51]. Cardiac dysfunction usually occurs as a consequence of inflammatory, oxidative stress, and apoptosis mechanisms mediated by MAPK signaling pathways, and ERK, JNK, and p38-MAPK activation has been demonstrated in heart failure models [51]. Furthermore, the effects of DZN, phenyl-saligenin-phosphate (PSP), and CPF and their toxic metabolites (diazoxone and CPF-oxone) on H9c2 cardiomyoblasts were investigated. Forty-eight h after exposure to DZN, diazoxone, or CPF-oxone (200 μM), no cytotoxicity was found in H9c2 cells [51]. However, CPF induced cardiotoxicity in H9c2 at a dose of over 100 μM. Phenyl-saligenin phosphate (PSP) induced cytotoxicity in mitotic and differentiated H9c2 cells by activating JNK1/2 signaling but not ERK1/2 signaling, suggesting the proapoptotic role of PSP for the induction of cell death [51].

## 7. Organophosphorus Compound-Induced Respiratory Diseases are Mediated by MAPK Signaling

Chronic obstructive pulmonary disease (COPD) is one of the most important respiratory diseases in which an increase in activated MAPK signaling is found in bronchial epithelial cells, alveolar macrophages, and pulmonary lymphocytes. Further, activated p38-MAPK is involved in local and systemic inflammation in COPD [52]. It has been reported that cell-based defense mechanisms mediated by dendritic cells play a major role in respiratory immunology and that dendritic cells potentially act on the first immune response in the respiratory system [52]. They further indicated that exposure to dimethoate or CPF reduced phosphorylation of Akt 1, Akt 2, Akt, and ERK 2 in human dendritic cells. In this context, dimethoate significantly decreased phosphorylation of the heat shock protein (HSP 27) and CPF slightly decreased its activity. However, dimethoate or CPF had no effect on p38-MAPK or the JNK [52]. Protein kinases including Akt or ERK are responsible for cell survival, which could be inhibited by OPCs, leading to an increase in inflammatory cytokines such as IL-1β and IL-6 and causing pulmonary complications [52].

## 8. Organophosphorus Compound-Induced Hepatotoxicity is Mediated by MAPK Signaling

The activated MAPKs signaling contributes to hepatic metabolic processes. The liver modulates the metabolism of glucose and lipids. Pathophysiological conditions such as diabetes, obesity, and fatty liver disease associated with disturbed liver functions. In addition, stress stimulates the activation of hepatic MAPKs, which leads to a disorder of insulin action and lipid metabolism. In this context, OPCs can influence the balance of MAPK signaling pathways in liver metabolism and can contribute to metabolic disorder [53]. Further, treatment of HepG2 cells with enantiomers of isocarbophos (ICP) upregulated Bax expression and downregulated Bcl-2 expression, leading to apoptosis. It was found that (_-_)-ICP induces a change in the Bax/Bcl-2 ratio and hepatotoxicity by the sustained activation of JNK [53]. The addition of tributyl phosphate (TBP) and tris (2-butoxy ethyl) phosphate (TBEP) to HepG2 produced ROS overproduction and caused mitochondrial and p53-mediated apoptosis via activation of JNK and ERK1/2 pathways by TBP and activation of the JNK pathway by TBEP [54].

## 9. Organophosphorus Compound-Induced Nephrotoxicity is Mediated by MAPK Signaling

Recent studies have shown that the MAPK/ERK signaling pathways play a major role in nephrogenesis and lead to differentiation of the nephrogenic mesenchyme. Moreover, an imbalance in MAPK signaling pathways causes several illnesses, such as cancers [55]. Interestingly, it has been reported that the development of kidney cells is caused by MAPK signaling and that an imbalance in the MAPK signaling pathways, especially in the nephron precursors, leads to a disturbance in nephron differentiation [55]. In addition, Tris-(2-chloroethyl)-phosphate (TCEP) with 0.01 and 1 mg/L^−1^ significantly increased the phosphorylation of JNK and reduced the expression of Bcl-2 and ClAP-2. In fact, TCEP also increased the expression of caspase-3 and caspase-9 in primary cultured proximal renal tubule cells [55].

## 10. Organophosphorus Compound-Induced Reproductive Toxicity is Mediated by MAPK Signaling

A large body of evidence has demonstrated a link between the dysfunction of the MAPK signaling pathways and the dysregulation of reproductive function [56]. In addition, the toxic effect of OPs on the reproductive system was also determined. The toxic effect of CPF and the metabolites 3,5,6-trichloro-2-pyridinol (TCP) and CPF-oxone was investigated on human placental choriocarcinoma cells (JAR), and JAR cells exposed to CPF (3–250 µM for 24, 48, and 72 h) had reduced the cell viability in a dose-dependent manner [56]. Further, CPF caused the loss of mitochondrial potential, DNA fragmentation, and upregulation of FAS mRNA. In this context, the addition of JNK, MEK, and PI3K inhibitors could not affect cell death. However, the p38-MAPK inhibitor (SB202190) significantly increased cell death. The results showed that activation of the p38-MAPK signaling pathway had a protective effect against CPF-induced cytotoxicity, and the p38-MAPK inhibitor might influence the isoform of p38-MAPK and might contribute to the activation of the survival signaling pathways [57]. CPF and CPF-oxone activated ERK44/42 signaling in both wild-type (CHOK1) and human muscarinic receptor-expressing ovarian cells of the Chinese hamster (CHO-M2). CPF-Oxone increased the activation of the ERK 44/42 pathway in CHOK1 2- to 3-fold control cells, depending on dose and time. Further, CPF-Oxon activated ERK 44/42 signaling in CHOK1 cells by P13-K, PKC, and MEK [57]. The same group also showed that diacylglycerol (DAG) induced the activation of ERK 44/42 in the ovary of the Chinese hamster (CHOK1). Pretreatment of CHOK1 cells with CPF-oxone or a carbamate increased the effect of DAG on the activation of ERK44/42 in a dose- and time-dependent manner, suggesting that inhibition of DAG lipase activity by CPF-oxone altered DAG metabolism and induced activation of the ERK 44/42 signaling pathway [58]. In another study by Lim et al., the effects of trichlorfon on the proliferation and apoptosis of porcine trophicotoderm (pTr) and uterine luminal epithelial cells (pLE) were investigated [59]. Trichlorfon prevented the proliferation of pTr and pLE cells, which led to apoptosis, loss of mitochondrial membrane potential, and DNA fragmentation. In addition, trichlorfon reduced the phosphorylation of pTr and pLE cells. Trichlorfon temporarily stimulated several molecular signaling proteins such as P70S6K, P90RSK, S6, JNK, and p38-MAPK, and inhibition of the ERK1/2, JNK, and p38-MAPK signaling pathways by trichlorfon reduced proliferation compared to each inhibitor alone in pTr cells. In addition, inhibition of ERK1/2 and p38-MAPK reduced proliferation of pLE cells, and this reduction was more pronounced when trichlorfon was combined with U0126 (ERK-inhibitor) or p38-MAPK inhibitors. The results indicated that trichlorfon had a synergic antiproliferative effect with the MAPK inhibitor on pLE and pTr cells during early pregnancy [59].

## 11. Organophosphorus Compound-Induced Cancer is Mediated by MAPK Signaling

It has been reported that OPCs can affect the immune system, which in turn can alter the differentiation and subversion of abnormal cells, thereby assessing the risk of cancer. Natural killer (NK) cells are an important component of the immune system and can interfere with tumor- and virus-infected cells, and the risk of cancer and infection can increase if the function of NK cells is impaired [60]. In this context, OPCs can disrupt the function of NK cells by activating MAPKs. Suriyo et al. investigated the inhibition of colorectal adenocarcinoma H508 cells with dose-dependent CPF (5–100 µM) treatment and found that the EGFR/ERK1/2 signaling pathway contributes to CPF-induced growth of colorectal adenocarcinoma H508 cells [60]. In addition, CPF (50 µM) caused cell death in human breast cancer cell lines MDA-MB-231 and MCF-7 by the altered redox imbalance mediated by ERK1/2 phosphorylation and the antioxidant system [61]. In another study by Park et al. [62], CPF-induced ROS production caused damage to the mitochondria and led to the swallowing of mitochondria in autophagous double-membrane vesicles. In addition, CPF caused a stabilization of protein kinase1 (PINK1) on the outer mitochondrial membrane, which led to an increase in parkin recruitment from the cytoplasm into the abnormal mitochondria, suggesting that PINK1 stabilization was modulated by ROS-induced JNK and ERK1/2 activation. In this context, DZN (10^−4^ to 10^−5^ M) for 24 h caused time-dependent cell death and reduced phosphorylated ERK2 in a dose-dependent manner, but phosphorylation of Raf-1, an upstream ERK molecule in the Ras-ERK cascade, could not affect cell death time [63]. Indeed, exposure of SH-SY5Y cells to non-neuropathic OPCs (paraoxone; 100 µM) increased MAPK activity while neuropathic OPCs (PSP; 0.01, 0.1, and 1.0 µM) inhibited activation of this pathway for 4, 8, 24, and 48 h [64].

## 12. Organophosphorus Compounds-Induced Hyperglycemia are Mediated by MAPK Signaling

It is known that environmental pollutants are the main factors involved in diabetes and its complications. Studies have shown a link between exposure to OPCs and the occurrence of diabetes [65]. Furthermore, Yuan et al. [65] investigated the effects of omethoate (0.5, 1, and 2 mg/kg, PO, 60 days) on liver insulin signaling in 60 male male mice. It was observed that a high dose of omethoate increased the expression levels of both NF-кB and p38-MAPK and that its mean dose increased the expression of p38-MAPK. Additionally, Zhang et al. pointed out that omethoate (1.5, 3, and 6 mg/kg body through the stomach for 2 months) increased MDA, IL-6, and TNF-α levels and decreased SOD and GPx activities in the right thigh muscles of rats by activating JNK, p38-MAPK, and master transcription factor NF-κB in the right thigh muscles, leading to insulin resistance. ERK1/2 is a major factor involved in insulin signaling, and an increase in these levels disrupts the insulin signaling pathway [66]. In a study by Mense et al., CPF and cyfluthrin significantly increased the levels of activated ERK1/2 in primary human fetal astrocytes, suggesting that CPF and cyfluthrin increased the inflammation markers IL-6 and GFAP similar to the signaling component ERK1/2 [67].

## 13. Organophosphorus Compound-Induced Dyslipidemia is Mediated by MAPK Signaling

It has been reported that the effect of OPCs on lipid metabolism is controversial. In this context, exposure to DZN disturbed lipid metabolism by altered MAPK signaling pathways. In addition, subacute DZN (15 mg/kg, PO) on the regulation of lipid metabolism, ERK, and expression of the low-density lipoprotein receptor (LDLr) in rat liver was evaluated. DZN significantly increased cholesterol, triglyceride, and LDL levels. Additionally, DZN reduced the activated ERK1/2 and LDLr transcripts, suggesting that DZN caused dyslipidemia and increased the expression of LDLr by inhibiting the ERK signaling pathway [68].

## 14. Conclusions

**Figure 1:**
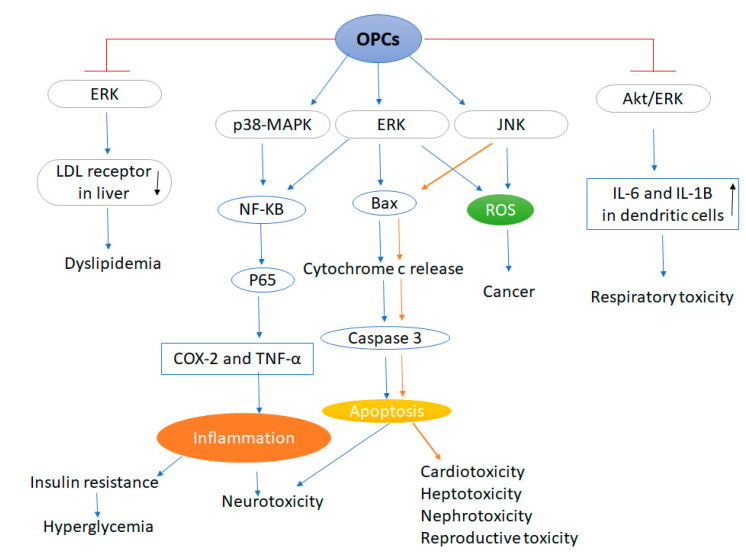
Organophosphorus compounds induced various diseases mediated by MAPK signaling. Abbrivations: OPCs: Organophosphate compounds, ERK: Extracellular signal-regulated protein kinase, LDL: Low density lipoprotein, MAPKs: Mitogen-activated protein kinases, NF-Κβ: Nuclear transcription factor kappa-β, COX-2: Cyclooxygenase-2, TNF-α: Tumor necrosis factor alpha, ROS: Reactive oxygen species, Akt: Protein kinase B, JNK: c-Jun NH2-terminal kinase. Blue arrows indicate the association between OPCs exposure, MAPK signaling and neurotoxicity, hyperglycemia and dyslipidemia. (↑): Increase, (↓): Decrease. Brown arrows indicate the association between OPCs exposure, MAPK signaling and cardiotoxicity, hepatotoxicity, nephrotoxicity and reproductive toxicity.

It is known that exposure to OPCs causes apoptosis through activation of ERK, JNK, and p38-MAPK signaling pathways. The members of the MAPK family appear to be affected differently by each OPC, depending on the cell type. In addition, MAPKs protect cell survival or induce equilibrium and can distinguish cell death between antiapoptotic and proapoptotic signals transmitted by ERK, JNK, and p38-MAPK signaling pathways (Figure 1).

The present review suggests that OPCs may induce apoptosis by interrupting the modulation of the MAPK signaling pathway. The activation of MAPKs in the target organs has a significant effect on the toxicity of OPCs. These compounds induce apoptosis and oxidative stress by disrupting the downregulation of Nrf2 and the equilibrium of phosphorylation of Erk, JNK, and p38-MAPK in tissues from animal and in vitro models. In addition, Erk, JNK, and p38-MAPK have pro- and antiapoptotic effects triggered by OPC-induced tissue damage. Moreover, MAPK signal transduction has both inhibitory and stimulatory effects on apoptosis, depending on cell type, stimulating factor, and latency of MAPK signaling activation. In particular, MAPK has a modulating effect on numerous downstream molecules, including transcriptional and translational components, cell cycle molecules, kinases leading to cell proliferation, cell cycle arrest, migration, differentiation, and apoptosis. These multiple downstream targets, cell type, and cell cycle-specific molecular cross-talk or kinetics and time of activation can cause the complexity of MAPK effects on apoptosis. Moreover, their balance ultimately determines the fate of the cells. However, the new findings could also clarify the controversial data on the various effects of OPCs, including their effect on blood glucose and lipid profile obtained in different cell types and animal models exposed to different OPCs.

It was found that some antioxidants with beneficial effects against toxic damage induced by OPCs could regulate the MAPK signaling pathways. Chemical antioxidants such as vitamin C and E, melatonin, N-acetylcysteine, etc. as well as flavonoids including curcumin, quercetin, berberine, etc. may be able to combat against OPC toxicity by modulating MAPK signaling.

In summary, the effect of OPCs in the initiation and progression of various diseases may be related to their effect on the regulation of MAPK signaling pathways. Studies need to be performed to determine the effect of the antidote of OPCs on MAPK signaling to find more suitable therapeutic approaches.

## Figures and Tables

**Table 1:** Organophosphorus compounds induced several diseases mediated by Mitogen-activated protein kinase (MAPK) signaling.

References	OPC(s)/Dose	Experimental Model	Findings
[30]	Monocrotophos; 10, 100, and 1000 μM	Human Cord Blood Mesenchymal Stem Cells	Increased ROS production through the activation of the ERK/AP-1 pathway
[32]	Chlorpyrifos; 0.75 ppm (diluted in 1% sucrose solution)	Drosophila flies	Increased the phosphorylation of p38 and JNK; no changes in the content of total forms of p38 and JNK
[34]	Chlorpyrifos; 0–200 μM; 0–24 h	SH-SY5Y cells	Induced cell apoptosis via activation of p38, JNK, and ERK
[36]	Phoxim; 4 mg/L added to the diet	Silkworms	Upregulated MAPK and PI3K/Akt signaling pathway genes
[37]	Diethyldithiophosphate; 1–50 μM	Human CD4+ T lymphocytes	Stimulated the activation of ERK, JNK, and p38 and NFAT nuclear translocation, leading to a decrease in cell proliferation
[38]	Chlorpyrifos; 0, 25, 50, 100, and 200 μM	PC12 cells	Induced apoptosis via activating the p38, JNK, and ERK; activated caspase-3 and cleavage of PARP
[39]	Chlorpyrifos; 25, 50, or 100 µM	SH-SY5Y cells	Induced generation of ROS and activation of MAPKs via expression of phospho-Drp1
[5]	Chlorpyrifos; 100 µM	SH-SY5Y cells	Induced apoptosis by producing ROS and upregulating COX-2 mediated by JNK and p38 pathways, independent of the activation of ERK1/2 signaling
[40]	Chlorpyrifos; 10 µM	Rat hippocampal neurons	Phosphorylation of ERK1/2 during 96 h exposure; withdrawal after 48 h exposure caused inhibition of ERK1/2 activation, leading to the delayed cytotoxicity in primary rat hippocampal neurons
[41]	Chlorpyrifos; 0–80 µM	Primary cortical neurons from embryonic day 17 or neonates rats	Activation of the ERK1/2- and JNK-induced apoptosis; activation of the p38-MAPK prevented apoptosis
[42]	Chlorpyrifos; 5 mg/kg, daily	Substantia nigra (SN) in young adults at PND 11–14	Induced dopaminergic neuronal damage in SN following the inflammatory response activation through NF-kB p65 and p38- MAPK pathways in the nigrostriatal system
[43]	Sarin; 80 μg/kg	Wistar rats	In the first 6 h after exposure, fast elevation in the activity of ERK1/2 with no change in JNK that temporarily inhibited apoptosis
[44]	Sarin and Soman-like agents; 4.0 mg/kg body weight; Intravenous injection	Wistar rats	Neurotoxicity via activation of JNK following tyrosine kinase phosphorylation
[45]	Soman; intramuscular administration; 60 μg kg^−1^	Wistar rat cerebellum	Elevated the expression of activated p38-MAPK and c-myc at 14 days after poisoning; c-jun and elk-1 expressions did not change at 14 days after poisoning
[46]	Soman; intramuscular administration; 60 μg kg^−1^	Rat cerebellar Purkinje cells	Elevated the expression of phosphorylated p38-MAPK and c-myc at 14 days after poisoning; both activated elk-1 and c-jun expressions were not changed at 14 days after poisoning
[47] [48]	Mevinphos, bilateral injection, of 10 nmol	Rostral ventrolateral medulla (RVLM) of rats	No effect on ERK1/2 and the total amount of JNK, p38-MAPK, MAP2K4, and MAP2K6. Increased the phosphorylation of ERK1/2 in Thr202 and Tyr204 and JNK in Thr183 and Tyr185, of p38-MAPK in Thr180 and Tyr182, of MAP2K4 in Ser257 and Thr261, and of MAP2K6 in Ser207 and Thr211 in RVLM and also ATF-2 in Thr71 and of c-Jun in Ser73 death
[49]	Mevinphos; 10 nmol; injected bilaterally	Rostral ventrolateral medulla (RVLM) of rats during brain stem death	Stimulated the phosphorylation of ERK1/2 at Thr202 and Tyr204
[50]	Bis(pinacolyl methyl phosphonate); 600 µM	Cultured rat astrocytes	Induced ERK signaling cascade for the induction of mitochondrial vacuolation
[51]	Phenyl saligenin phosphate; 0–200 µM	Mitotic and differentiated H9c2 cardiomyoblasts	Induced cytotoxicity by activating JNK1/2 but not ERK1/2
[52]	Chlorpyrifos and dimethoate; 0–1000 µM	Human dendritic cells	Decreased the phosphorylation of Akt 1, Akt 2, Akt, and ERK 2 and caused pulmonary complications.; no effect on the p38 or the JNK
[53]	Enantiomers of isocarbophos; 0–40 µM	Human hepatoma cells	(−)-ICP caused modification in Bax/Bcl-2 ratio and hepatotoxicity via sustained activation of the JNK
[54]	Tributylphosphate and tris (2-butoxy ethyl) phosphate; 50, 100, and 200 μM	Human hepatoma cells	Induced mitochondrial and p53-mediated apoptosis via activated JNK and TBP also affected ERK1/2
[55]	Tris-(2-chloroethyl)-phosphate; 0.01 and 1 mg/L^−1^	Primary cultured renal proximal tubule cells	Elevated the phosphorylation of JNK
[56]	Chlorpyrifos; 3–250 µM for 24, 48, and 72 h	Human placental choriocarcinoma (JAR) cells	Activated the p38-MAPK signaling pathway protected against cytotoxicity
[57]	Chlorpyrifon-oxon; 50 µM for 40 min	Wild-type (CHOK1) and human muscarinic receptor-expressing Chinese hamster ovary cells (CHO-M2)	Activated the ERK 44/42 signaling through P13-K, PKC, and MEK
[58]	Chlorpyrifos-oxon; 0–100 µM	Chinese hamster ovary (CHOK1)	Increased the effect of diacylglycerol on ERK 44/42 activation in dose and time dependent manner
[59]	Trichlorfon; 100 µM	Porcine trophectoderm (pTr) and uterine luminal epithelial (pLE) cells	Temporarily activated JNK and p38-MAPK; inhibition of JNK, p38-MAPK, and ERK1/2 decreased the proliferation in pTr cells
[60]	Chlorpyrifos; 5–100 µM	Colorectal adenocarcinoma H508 cells	Caused colorectal adenocarcinoma H508 cell growth via involvement of EGFR/ERK1/2 signaling pathway
[61]	Chlorpyrifos; 50 µM	MDA-MB-231 and MCF-7 human breast cancer cell lines	Caused cell death through ERK1/2 phosphorylation-mediated
[62]	Chlorpyrifos; 0, 25, 50, and 100 µM	SH-SY5Y cells	Caused protein kinase 1 stabilization on the outer mitochondrial membrane; resulted in an elevation in Parkin recruitment from the cytoplasm to the abnormal mitochondria; PINK1 stabilization was modulated by ROS-mediated activation of JNK and ERK1/2 signaling.
[63]	Diazinon; 10^−4^ to 10^−5^ M	NT2 cells	Reduced the phosphorylated ERK-2 dose-dependently; phosphorylation of Raf-1 did not affect
[64]	Paraoxon: 100 µM; Phenyl saligenin phosphate: 0.01, 0.1, and 1.0 µM	SH-SY5Y cells	Paraoxon elevated the activity of the MAPK pathway; Phenyl saligenin phosphate inhibited the activation of the MAPK pathway
[65]	Omethoate; 0.5, 1, and 2 mg/kg, PO, 60 days	ICR male mice	At high doses, increased the expression levels of both NF-кB and p38 MAPK; at medium doses, increased the expression of p38-MAPK
[66]	Omethoate; 1.5, 3, and 6 mg/kg body through gastric for 2 months	Wistar rats	Increased the levels of MDA, TNF-α, and IL-6 and decreased the activities of SOD and GPx through activation of JNK, p38 MAPK, and NF-κB, leading to insulin resistance.
[67]	Chlorpyrifos and cyfluthrin; 0, 25, 50, and 100 µM	Primary human fetal astrocytes	Increased the levels of activated ERK1/2; increased inflammatory markers IL-6 and GFAP
[68]	Diazinon; 15 mg/kg, PO	Liver of rats	Induced hyperlipemia and increased levels of LDLr transcription through inhibition of ERK pathway

Abbrivations: Akt: Protein kinase B, AP-1: Activator protein 1, COX-2: Cyclooxygenase-2, Elk-1: ETS Like-1, ERK: Extracellular signal-regulated protein kinase, GFAP: Glial fibrillary acidic protein, GPx: Glutathione peroxidase, ICP: Isocarbophos, iNOS: inducible nitric oxide synthase JNK: c-Jun NH2-terminal kinase, LDLr: Lipoprotein receptor, MAPKs: Mitogen-activated protein kinases, MEK: Mitogen-activated protein kinase kinase, NF-Κβ: Nuclear transcription factor kappa-β, NO: Nitric oxide, PARP: Poly (ADP-ribose) polymerase, PI3Ks: Phosphoinositide 3-kinases, PKC: Protein kinase C, PKG: Protein Kinase G, PO: Per Os, PTr: Porcine trophectoderm, RVLM: Rostral ventrolateral medulla, Ser73: Phospho-c-Jun, SN: Substantia nigra, SOD: Superoxide dismutase, Thr180/Tyr182: Phospho-p38 MAPK, Thr261: Phospho-SEK1/MKK.

## Abbreviations

AChE	Acetylcholinesterase
Akt	Protein kinase B
AP-1	Activator protein 1
ASK1	Apoptosis-signal-regulating kinase 1
ATF2	Activating transcription factor 2
BPMP	Bis (pinacolylmethyl) phosphonate
CAT	Catalase
CHO-M2	Chinese hamster
COPD	Chronic obstructive pulmonary disease
COX-2	Cyclooxygenase-2
CPF	Chlorpyrifos
CREB	cAMP response element
DDVP	Dichlorvos
Drp1	Dynamin-related protein 1
DZN	Diazinon
Elk-1	ETS Like-1
ERK	Extracellular signal-regulated protein kinase
Fox	Forkhead box
GFAP	Glial fibrillary acidic protein
GPx	Glutathione peroxidase
GSH	Glutathione
HO-1	Heme oxygenase 1
HSP 27	heat shock protein
HUVECs	Human umbilical cord endothelial cells
ICP	Isocarbophos
IL-6	Interleukin-6
iNOS	inducible nitric oxide synthase
JNK	c-Jun NH2-terminal kinase
LDLr	Lipoprotein receptor
MAPKs:	Mitogen-activated protein kinases
MCP	Monocrotophos
MDA	Malondialdehyde
MEK	Mitogen-activated protein kinase kinase
Mev	Mevinphos
MSK1/2	Mitogen- and stress-activated protein kinases 1 and 2
NF-Κβ	Nuclear transcription factor kappa-β
NGF	Nerve growth factor
NO	Nitric oxide
NQO-1	NAD(P)H quinone oxidoreductase
OPCs	Organophosphate compounds
OPs	Organophosphate pesticides
p70S6K	phosphorylated-p70 ribosomal S6 protein kinase
PARP	Poly (ADP-ribose) polymerase
PI3Ks	Phosphoinositide 3-kinases
PINK1	Protein kinase1
PKC	Protein kinase C
PKG	Protein Kinase G
PLC-gamma	Phospholipase C gamma
PO	Per Os
PON1	Paraoxonase1
PP2C epsilon	Protein phosphatase 2C cDNA
PPAR-γ	Peroxisome proliferator-activated receptor-gamma
PSP	Phenyl-saligenin-phosphate
PTr	Porcine trophectoderm
RGZ	Rosiglitazone
ROS	Reactive oxygen species
RVLM	Rostral ventrolateral medulla
Ser73	Phospho-c-Jun
SN	Substantia nigra
SOD	Superoxide dismutase
TCEP	Tris-(2-chloroethyl)-phosphate
TCP	3,5,6-trichloro-2-pyridinol
TDCPP	Tris(1,3-dichloro-2-propyl)phosphate
Thr180/Tyr182	Phospho-p38 MAPK
Thr261	Phospho-SEK1/MKK
TNF-α	Tumor necrosis factor alpha
Trx	Thioredoxin
